# Weekly cisplatin, epirubicin, and paclitaxel with granulocyte colony-stimulating factor support *vs* triweekly epirubicin and paclitaxel in locally advanced breast cancer: final analysis of a sicog phase III study

**DOI:** 10.1038/sj.bjc.6603395

**Published:** 2006-10-17

**Authors:** G Frasci, G D'Aiuto, P Comella, R Thomas, G Botti, M Di Bonito, V De Rosa, G Iodice, M R Rubulotta, G Comella

**Affiliations:** 1 Giuseppe Frasci, Division of Medical Oncology A, National Tumor Institute, via Mariano Semmola 80131, Naples, Italy

**Keywords:** paclitaxel, epirubicin, cisplatin, weekly administration, LABC, randomised trial

## Abstract

The present study aimed at evaluating whether a weekly cisplatin, epirubicin, and paclitaxel (PET) regimen could increase the pathological complete response (pCR) rate in comparison with a tri-weekly epirubicin and paclitaxel administration in locally advanced breast cancer (LABC) patients. Patients with stage IIIB disease were randomised to receive either 12 weekly cycles of cisplatin 30 mg m^−2^, epirubicin 50 mg m^−2^, and paclitaxel 120 mg m^−2^ (PET) plus granulocyte-colony stimulating factor support, or four cycles of epirubicin 90 mg m^−2^+paclitaxel 175 mg m^−2^ (ET) every 3 weeks. Overall, 200 patients (PET/ET=100/100) were included in this study. A pCR in both breast and axilla occurred in 16 (16%) PET patients and in six (6%) ET patients (*P*=0.02). The higher activity of PET was evident only in ER negative (27.5 *vs* 5.4%; *P*=0.026), and in HER/neu positive (31 *vs* 5%; *P*=0.037) tumours. The two arms yielded similar pCR rate in ER positive (PET/ET=7.5/7.1%) and HER/neu negative (PET/ET=10/6%) patients. At a 39 months median follow-up, 70 patients showed a progression or relapses (PET, 32 *vs* ET, 38). Anaemia, mucositis, peripheral neuropathy, and gastrointestinal toxicity were substantially more frequent in the PET arm. The PET weekly regimen is superior to ET in terms of pCR rate in LABC patients with ER negative and/or HER2 positive tumours Mature data in terms of disease-free and overall survival are needed to ascertain whether this approach could improve the prognosis of these subsets of LABC patients.

The role of chemotherapy in improving the prognosis of locally advanced breast cancer (LABC) patients is widely recognised. High objective response rates can be achieved with standard combination chemotherapy, rendering in most cases the tumour operable at the end of the treatment (Hortobagyi and Buzdar, 1997). Primary chemotherapy is also becoming very popular in patients with operable disease at diagnosis, since it permits a substantial increase in the rate of breast-sparing surgery, and allows an early pathological evaluation of the effect of treatment. The absence of residual tumour in both breast and axilla is associated with a much lower risk of relapse (Chollet *et al*, 2002; Faneyete *et al*, 2003). Unfortunately, the chance of achieving such a goal in women with LABC is low. Indeed, in the NSABP-B18 trial, the pCR rate after primary chemotherapy was <3% in women with T3 (operable) disease (Fisher *et al*, 1997). During the last decade, several new drugs with different mechanisms of action have been introduced into clinical practice. Among them, the taxanes (paclitaxel and docetaxel) have raised great enthusiasm, and have been rapidly incorporated in combination regimens (Dombernowsky *et al*, 1996; Moliterni *et al*, 1997).

Phase III trials comparing taxane- *vs* non taxane-including regimens, have been carried out in breast cancer patients with loco-regional disease. Both in the NSABP-B27 (Bear *et al*, 2003) and in the Aberdeen (Smith *et al*, 2002) studies, the addition of docetaxel resulted in a two-times greater pCR rate in comparison with the standard arm. Notably, a substantial proportion of patients in the Aberdeen trial had LABC. French investigators (Dieras *et al*, 2004) reported a significantly higher pCR rate with primary doxorubicin plus paclitaxel in comparison with a standard doxorubicin plus cyclophosphamide regimen.

A better therapeutic index has been recently hypothesised for the weekly paclitaxel administration. In an MDACC trial (Green *et al*, 2005), 258 patients with operable disease were randomised to receive either weekly or every 3 weeks paclitaxel followed by four FAC (5-fluorouracil, doxorubicin and cyclophosphamide cycles before surgery. The pCR rate in the weekly arm was twice that of the tri-weekly arm (28.8 *vs* 13.6%).

In the last few years, increasing interest has arisen about the role of platinum compounds in the treatment of breast cancer patients. Several cisplatin-based regimens have been tested in the neo-adjuvant setting, showing high anti tumour activity (Orlando *et al*, 2001; Ezzat *et al*, 2004). In the mid-nineties, we started the assessment of a weekly triplet regimen including cisplatin, epirubicin, and paclitaxel (PET regimen) in breast cancer patients (Frasci *et al*, 1999). This weekly approach was initially tested in a phase II study in women with LABC (Frasci *et al*, 2000). The promising activity data we observed (overall response rate, 90%, and pCR rate, 12%) prompted us to start the present phase III trial, which aimed at comparing this regimen with a tri-weekly epirubicin plus paclitaxel administration.

## METHODS

### Eligibility criteria

Patients with histological proven locally advanced (T4 a-d and/or N2 fixed to other structures) breast cancer with no prior chemotherapy were eligible. Other selection criteria were: age <75, ECOG performance status ⩽2, adequate bone marrow (ANC ⩾2.0 × 10^9^ l^−1^, platelet count ⩾100 × 10^9^ l^−1^, and haemoglobin level ⩾100 g l^−1^), liver (bilirubin level <1.5 the upper normal limit [UNL], AST and/or ALT <3 × UNL, prothrombin time <1.5 times control), renal (creatinine clearance ⩾60 ml min^−1^) and cardiac functions (left ventricular ejection fraction >50%, absence of severe cardiac arrhythmia or heart failure, second- or third-degree heart block or acute myocardial infarction within 4 months prior to study entry). Previous or concurrent malignancy, were also considered as exclusion criteria, except for inactive nonmelanoma skin cancer, and *in situ* carcinoma of the cervix. All patients gave their written informed consent, and the trial was approved by the Independent Ethical Committee of the National Tumour Institute of Naples.

### Pretreatment evaluation

Within 4 weeks before starting chemotherapy, all patients underwent the following studies: complete history and physical examination, ECG and bi-dimensional echocardiography, mammography, chest X-ray, liver ultrasonography, radionuclide bone scan (with X-ray evaluation of suspicious bone segments), and CT or MRI of the brain in the case of suspected brain involvement. Laboratory investigation included a complete blood cell count with white blood cell (WBC) differential and platelet count, BUN, creatinine, bilirubin, SGOT, alkaline phosphatase, lactate dehydrogenase, prothrombin time, partial thromboplastin and thrombin time, urinalysis. A core biopsy of the primary tumour was also performed with the immunohistochemical assessment of the main prognostic variables (steroid hormones receptors, Ki67, HER2/neu). The HER2/neu was measured by using the monoclonal antibodies Mab 1 and CB11 on 3 *μ*m sections of paraffin-embedded tumour samples.

### Treatment

All eligible patients were randomly allocated to receive: (arm A) epirubicin 50 mg m^−2^ as an i.v. bolus, followed by paclitaxel 120 mg m^−2^ as a 1-h infusion, and cisplatin 30 mg m^−2^ as a 30 min infusion, weekly for a maximum of 12 cycles. Recombinant human G-CSF 300 *μ*g day^−1^ was also given subcutaneously on days 3–5 of each week. Short-term forced hyperhydration (1 l of saline over 2 h), and prophylaxis for nausea/vomiting (HT3 receptor antagonists) were also performed. Prophylaxis for hypersensitivity reactions consisted of dexamethasone 8 mg i.v. and promethazine 50 mg i.m. plus ranitidine 50 mg i.v. 30 min before paclitaxel administration; or (arm B) epirubicin 90 mg m^−2^ as an i.v. bolus, followed by paclitaxel 175 mg m^−2^ as a 1-h infusion, every 3 weeks for a maximum of four cycles. The same antiemetic and antihypersensitivity procedures adopted for arm A were performed.

Within 4 weeks from the end of chemotherapy patients underwent surgery. Breast sparing surgery was performed whenever feasible. It consisted of quadrantectomy together with standard level I and II axillary lymph-node dissection.

Four cycles of CMF were delivered after surgery, in patients whose pathologic assessment showed less than four involved axillary lymph nodes; whereas four FEC (epirubicin 60 mg m^−2^ instead of methotrexate) cycles were administered in those women showing four or more involved axillary nodes. On completion of postoperative chemotherapy, radiotherapy was performed on all patients who underwent conservative surgery, and to those who underwent mastectomy and had more than three axillary nodes involved, muscle, skin and/or nipple involvement, or had G3 tumour at diagnosis.

Hormone treatment was also given on completion of postoperative chemotherapy. LH-RH analogue for 2 years together with tamoxifen for 5 years was administered in premenopausal, and 5-year tamoxifen was given in postmenopausal women.

## DOSE ADJUSTMENTS ACCORDING TO TOXICITY

Arm A: chemotherapy was given at full doses if neutrophil count was ⩾1.5 × 10^9^ l^−1^, and platelet count was ⩾100 × 10^9^ l^−1^. Doses were reduced by 50% if neutrophil count was < 1.5/10^9^ and ⩾1.0/10^9^ l^−1^, or platelet count was <100 × 10^9^ l^−1^ and ⩾75/10^9^ l^−1^. In the case of grade ⩾3 neutropenia, grade ⩾2 thrombocytopenia, or grade >1 nonhaematological toxicity the treatment was always omitted. In the presence of grade 4 neutropenia, febrile neutropenia, grade 4 thrombocytopenia, grade 4 anaemia, grade 3–4 nonhaematological toxicity (except for alopecia) doses were reduced by 25% in the subsequent administrations. G-CSF was allowed in the presence of grade 4 neutropenia of any duration, or neutropenic fever, or if grade 3 neutropenia persisted for 2 weeks after the scheduled time of recycling. The treatment was definitively discontinued if chemotherapy could not be delivered 3 weeks after the scheduled time of recycling.

Arm B: chemotherapy was given at full doses for neutrophil count ⩾2000 mm^−3^, and platelet count ⩾100 000 mm^−3^. If neutrophil count was ⩾1500 mm^−3^ persisted after a 1-week delay, chemotherapy was delivered at 75% of the planned dose.

In the presence of grade 4 neutropenia, febrile neutropenia, grade 4 thrombocytopenia, grade 4 anaemia, grade 3–4 nonhaematological toxicity (except for alopecia) doses were reduced to 75% in the subsequent administrations. G-CSF was allowed in the presence of grade 4 neutropenia of any duration, or neutropenic fever, or if grade 3 neutropenia persisted for 2 weeks after the scheduled time of recycling. The treatment was definitively discontinued if chemotherapy could not be delivered three weeks after the scheduled time of recycling.

### Toxicity and response evaluation criteria

Toxicity was assessed at the weekly visits and recorded using the NCI-CTC version 3.0. Complete blood cell and WBC count were performed once a week in all patients, and every other day in cases of grade 4 haematological toxicity. Bidimensional echocardiography was performed on completion of treatment.

Clinical tumour response was assessed within 2 weeks from the end of chemotherapy, and classified according to standard WHO criteria (Miller *et al*, 1981). Clinical examination, mammography, breast ultrasonography were performed to assess the regression of the tumour in both breast and axilla. In addition, chest X-ray, and abdomen ultrasonography were also performed, in order to exclude the presence of distant metastases.

For pathologic assessment of response, the amount of residual epithelial neoplastic cells in the tumour mass, and the location of malignant component (invasive *vs* intraductal) were taken into account (Chevallier *et al*, 1993). Response in the breast was scored as follows: class I (absence of residual malignant epithelial cells), class II (persistence of only *in situ* residual malignant component), class III (only focal invasive tumour residuals), class IV (no substantial modifications in the tumour mass). Patients showing a class I or II response in the breast, together with absence of axillary involvement were considered as pCR.

### End points and statistical considerations

The pCR rate was the main end point. We set as 5% the pCR rate expected with the administration of epirubicin plus paclitaxel, and we hypothesised a 10% increase with the PET regimen. To have an 80% power to demonstrate such a difference with an alpha error <5%, at least 90 patients per arm were required (Casagrande *et al*, 1978).

Progression-free survival (PFS) and overall survival (OS) were also analysed. PFS was considered as the interval between randomisation and documentation of progressive disease or relapse (in surgically treated patients). Overall survival was considered as the interval between randomisation and death by any cause. All time-dependent curves were estimated by the Kaplan Meier method (Kaplan and Meier, 1958). All patients were included in the analysis of response and survival on an ‘intent to treat basis’.

## RESULTS

### Demographics

Between May 1999 and November 2004, 200 consecutive women with LABC were enrolled into this study. Table 1 outlines the main patient characteristics. Overall, 136 patients had T4a-b-c disease, while 47 women had inflammatory carcinoma, and 17 N2 disease. Patients (144) had ductal carcinoma, and oestrogen or progesterone receptors were positive in 110 and 103 patients, respectively. Patients (43) were HER2+.

### Compliance to proposed treatment

Women (13) in the PET and seven women in ET arm did not complete the planned number of chemotherapy cycles. Early disease progression was the cause of treatment discontinuation in three PET and four ET patients. The remaining early discontinuations were a consequence of severe toxicity or patient's refusal: in the PET arm, reasons for withdrawal were: severe emesis in two cases, persistence of severe mucositis in one case, severe peripheral neuropathy in two cases, severe fatigue in three cases, and patient's refusal in two cases. In the ET arm, severe emesis (one case), and fatigue (two cases) forced the treatment interruption.

**Figure 1:**
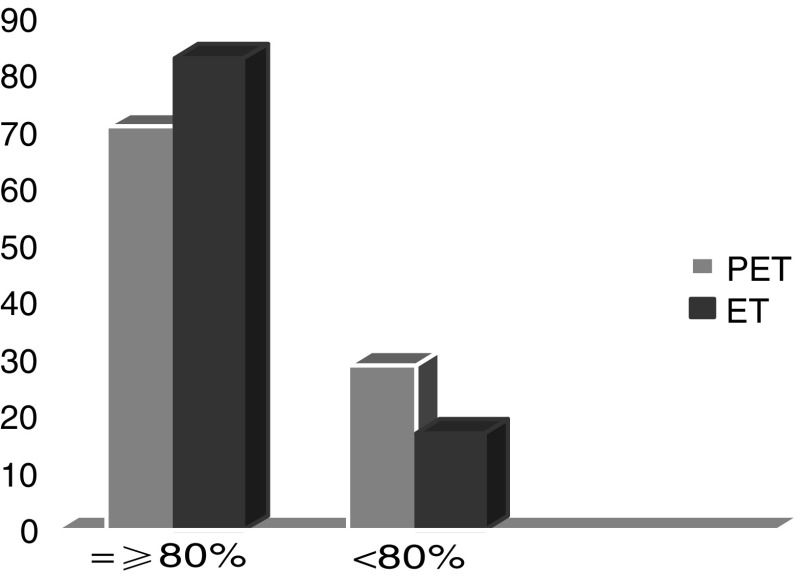
Relative dose Intensity.

A treatment delay due to persistence of haematological toxicity or grade >1 non haematological toxicity on day of recycling occurred in 18 and 11 patients in PET and ET arm, respectively. A dose reduction was performed at least once in 35 PET and 21 ET patients, respectively. In all, 71 PET and 83 ET patients actually received ⩾80% of the planned dose-intensity (Figure 1).

### Response

All the 200 enrolled patients were included into the response analysis on an ‘intent to treat basis’. Table 2 summarises clinical response data. In the PET arm, 28 complete and 60 partial responses were recorded at clinical restaging. An additional nine patients showed a minor regression or stabilisation of the tumour. In the ET arm, a clinically complete or partial response occurred in 19 and 59 patients, respectively. An additional 18 women showed a minor response or no change.

According to receptor status, a clinical complete response occurred in 19/40 (47.5%) ER negative and 8/53 (15%) ER positive patients (one CR in a woman with hormone receptor status unknown) in the PET arm. In the ET arm, a clinical complete response occurred in 6/37 (16.2%) ER negative, and 12/57 (21%) ER positive patients (one CR in a patient with unknown hormone receptor status). Regarding HER2/neu status, 9/23 (40%) HER2+ and 16/68 (23.6%) HER2− achieved a complete response in the PET arm, as compared to 7/20 (35%) HER2+ and 10/68 (14.7%) HER 2− in ET arm (Table 3).

Overall, 186 women (PET 94, ET 92) underwent surgery. Breast sparing surgery was performed in a total of 37 patients (PET 23, ET 14). Table 4 summarises the pathological response data. In all, 22 patients (PET 13, ET 9) showed absence of residual malignant epithelial cells, either invasive or intraductal, in the breast specimen. Only *in situ* residual tumour cells were found in additional 14 women (PET 9, ET 5). Therefore, the pCR rate in the breast was 22% in PET and 14% and ET arm, respectively. A pPR in the breast (i.e., only focal invasive tumour residuals in the removed breast tissue) was recorded in 38 PET and 26 ET patients, respectively. Overall, 59 women (PET 35, ET 24) showed negative axilla. Among the 141 patients with persistence of tumour in the axilla, 60 (PET 33, ET 27) had one to three lymph nodes involved, and 81 (PET 32, ET 49) four or more nodes.

In all, 22 patients (PET 16, ET 6) showed an absence of invasive tumour in both breast and axilla; the pCR rate being 16 and 6% in PET and ET arm, respectively (*P*=0.02). In the PET arm, 11 pCRs were observed among 40 ER negative tumours (27.5%) as compared to four pCRs among 53 ER positive (7.5%), and one among the seven with unknown hormone receptor status (14.3%). In the ET arm, two pCRs were registered in the 37 ER negative (5.4%) and four pCRs in the 57 ER positive tumours (7.1%). One out of four women with ER positive tumour, achieving a pCR, had negative PgR. All 11 ER negative tumours who achieved a pCR in the PET arm, were PgR negative too. If a comparison between the two arms is made taking into account the hormone receptor status, the advantage of PET treatment is highly evident in ER negative patients (PET 27.5 *vs* ET 5.4%; *P*=0.026), while it did not appear in the ER positive subgroup (PET 7.5 *vs* ET 7%) (Table 5).

Eight pCRs were observed in the 43 patients (18.6%) HER2/neu positive, as compared to 11/136 (8%) HER2 negative cases (3/21 patients with HER2/neu unknown had a pCR). Also in this case the superiority of the PET regimen was evident only in those patients with HER2 positive tumour (31 *vs* 5%; *P*=0.037) (Table 5).

**Figure 2:**
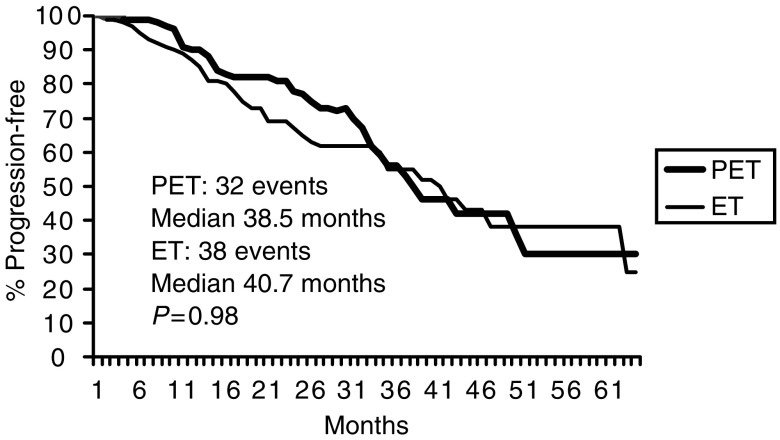
Progression free survival.

After a median follow-up of 39 (range, 4–70) months, 70 patients had progressed or relapsed (PET 32, ET 38), and the median PFS was 38.5 and 40.1 months in PET and ET arm, respectively. Five-year projected PFS probability was 30 and 25% for PET and ET, respectively (Figure 2).

**Figure 3:**
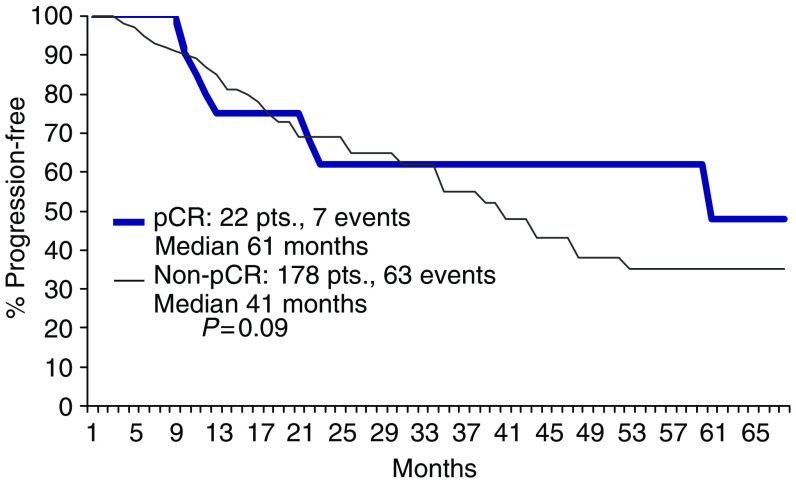
DFS according to pathological response.

Seven (PET 4, ET 3) of the 22 patients showing a pCR had relapsed at the time of the present analysis (Figure 3).

**Figure 4:**
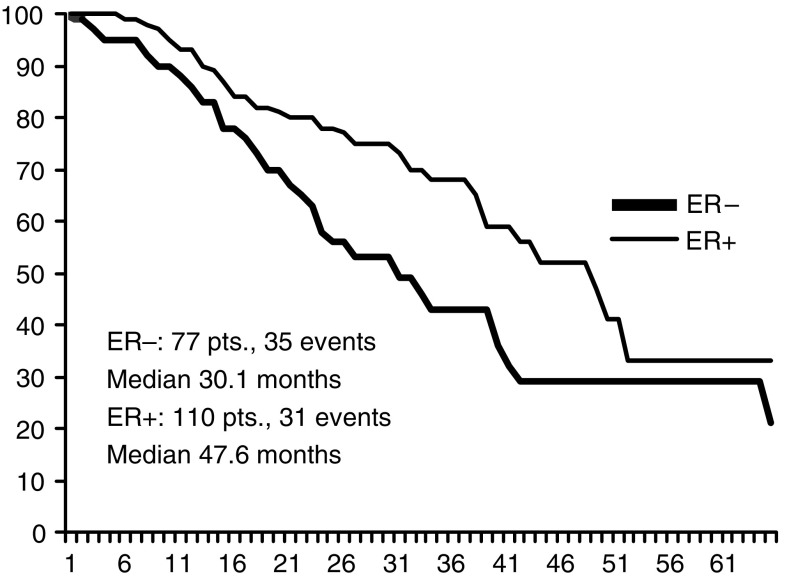
PFS according to ER status.

**Figure 5:**
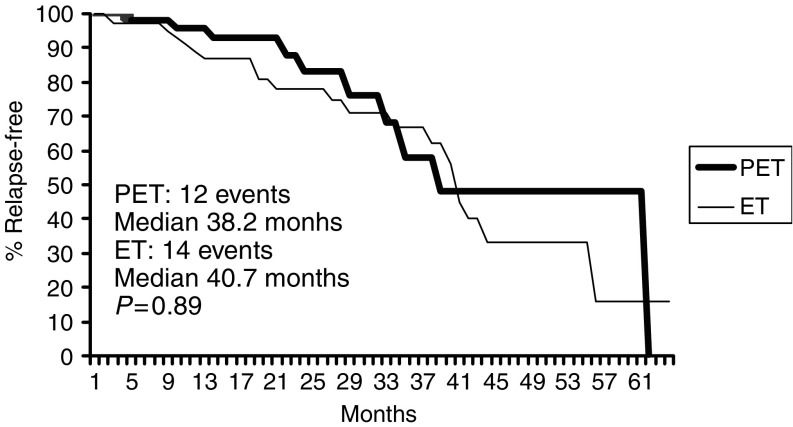
PFS in ER/PgR positive.

**Figure 6:**
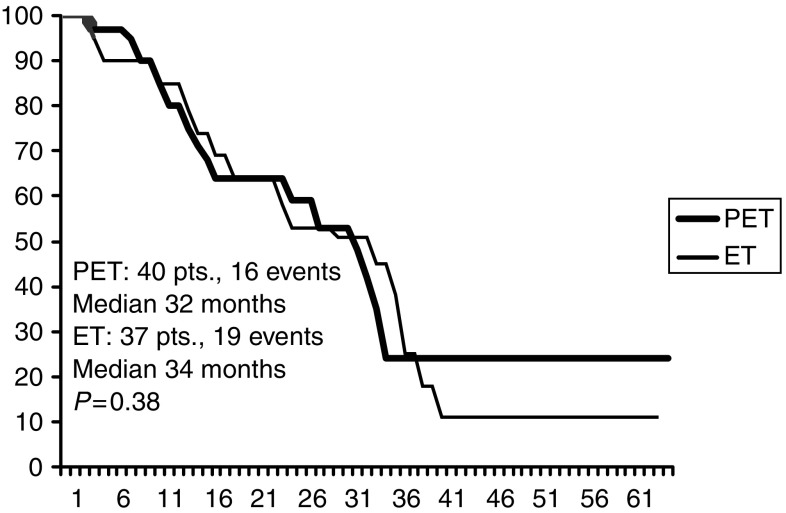
PFS in ER negative patients.

Overall, 35 out of 77 patients with ER negative tumour (PET 16, ET 19) had progression or relapse at the time of the present analysis, as compared to 31 (PET 14, ET 17) out of 110 patients with ER positive tumour; median PFS being 30.1 and 47.6 months, respectively (Figure 4). Median PFS times in ER positive patients were 38.2 months and 40.7 months for PET and ET, respectively; while they were 32 and 34 months, respectively, in ER negative patients (Figures 5, 6). Four failures occurred in the 13 patients with unknown receptor status.

**Figure 7:**
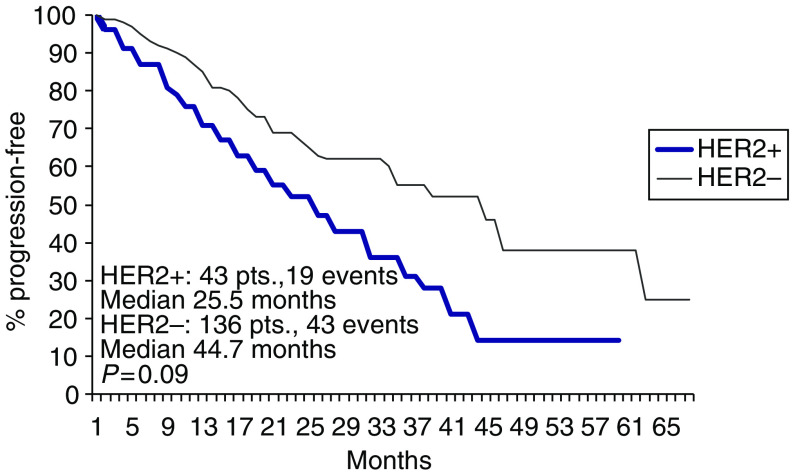
PFS according to HER2 status.

Overall, 19 (PET 8, ET 11) of the 43 patients with HER2 positive tumour had progressed or relapsed, median PFS being 25.5 months. A total of 43 (PET 20, ET 23) failures had occurred in the 136 HER2 negative patients, median PFS being 44.7 months (Figure 7).

Overall, 26 deaths (PET 10, ET 16) had occurred at the time of the present analysis. Median survival had not been reached in both arms.

### Toxicity

A total of 1122 PET courses and 386 ET courses were delivered and analysed for toxicity. No toxic deaths were observed in either arms. Table 6 shows the number of patients in each arm who experienced at least one grade ⩾3 toxicity.

Haematological toxicity was not substantially different in the two arms, except for anaemia. Overall 45 patients in PET and 38 in ET arm experienced a grade ⩾3 neutropenia. Grade 4 occurred in 17 and 18 cases, respectively. Neutropenic fever was observed in three PET and four ET patients. Severe thrombocytopenia was almost anecdotic, grade 3 or 4 occurring in only six patients (four PET and two ET). Only one case of grade 4 thrombocytopenia was recorded, occurring in the PET arm. Severe anaemia occurred in one ET patient, as compared to 13 (13%) PET patients.

Overall, severe gastrointestinal toxicity and fatigue were also more frequent in the PET arm. They caused early chemotherapy discontinuation in a total of eight patients (five PET and three ET). Severe emesis, loss of appetite, and diarrhoea occurred in 10, 13, and 15% of PET as compared to 4, 5, and 2% of ET arm. Severe fatigue occurred in 11 (11%) patients of PET and two (2%) patients of ET arm.

Neurotoxicity and mucositis and were also common side effects in both arms. Overall, 34 (34%) PET and 16 (16%) ET patients complained of sensory neuropathy, but it was severe in only three patients of PET arm, and in two cases it caused treatment discontinuation. Severe mucositis was recorded in 12 PET patients, and in one case it caused the definitive treatment discontinuation. Only one patient of the ET arm experienced severe mucositis. Renal, liver and cardiac toxicity were very uncommon. There were no cases of severe functional impairment of these organs. Five PET and three ET patients had a LVEF decrease below 50%. In all but one patient (treated with PET), LVEF returned within the normal range. No patients showed clinical signs of congestive heart failure.

## DISCUSSION

In the present randomised study, we aimed at evaluating whether 12 weekly preoperative cycles of PET with G-CSF support could improve the pCR rate achievable in patients with LABC (stage IIIB) in comparison with ET tri-weekly administration.

Our results seem to suggest that the PET weekly treatment is more effective than the tri-weekly ET regimen in producing a pCR. Indeed, the chance of achieving a complete clearance of the tumour in both breast and axilla in the PET arm was more than twice that of the ET arm.

The rate of pCR obtained in the present study with the standard treatment (ET) does not seem to be underestimated. In the Milan NCI experience, the use of neoadjuvant doxorubicin-paclitaxel combination gave a 7% pCR rate in a study population including 41 II/IIIA and 38 IIIB patients (Moliterni *et al*, 1997). Canadian authors recently reported a 9% pCR rate in 49 patients with locally advanced disease, 60% of whom had stage IIB and IIIA. They also remarked that, depending on the definition of pCR used, the range of pCR varied from 4.2% (using definition from Chevallier) to 10.6% (using that of NSABP-27 study) (Dent *et al*, 2005).

The present study was sized setting the pCR rate as the target end point. Of course, the achievement of a complete eradication of the tumour in both breast and axilla may be considered a meaningful end point only if it is associated with a substantial improvement of the long-term disease-free survival.

At the time of the present analysis, the number of observed failures (70 progressions or relapses) is too low to even allow us to speculate about the impact that this new regimen may have on prognosis of these patients. Moreover, given the very small rate of patients who are expected to have a substantial DFS gain (16 *vs* 6% pCRs), a much larger study population would be required to statistically detect such a gain.

Analysis of our data strongly suggests that this dose dense approach may be superior only in some subsets of patients and not in the whole population. Indeed, an impressive increase of the pCR rate with the PET regimen was obtained in patients with ER negative (27.5 *vs* 5.4%) and/or HER2/neu positive tumour (31 *vs* 5%), while the activity of the two regimens was comparable in patients with ER positive (7.5 *vs* 7%) and/or HER2/neu negative tumour (10 *vs* 6%). The modest effect of the primary chemotherapy in ER positive tumours was noted by other investigators, who recently reported a much higher pCR rate with chemotherapy in ER/PgR negative patients (Faneyete *et al*, 2003; Colleoni *et al*, 2004; Ring *et al*, 2004; Gianni *et al*, 2005) and seems to suggest that aggressive chemotherapy should not be advisable in hormone-sensitive tumours. In a previous phase II study, testing the PET regimen in patients with large operable breast cancer, we observed a pCR rate in 66% in ER negative patients as compared to 14% in ER positive (Frasci *et al*, 2005).

In the present study, seven out of 22 pCRs relapsed. This relapse rate looks similar to that of the total population. However, in the ER/PgR negative group 4/13 pCRs relapsed as compared to 31/64 non-pCRs. Moreover, three out of four relapsing pCRs were also HER2 positive, and in two cases brain was the first site of relapse. These findings suggest that chemotherapy alone is not enough in the management of HER2 positive patients, who might benefit of a prolonged trastuzumab treatment.

On the other hand, the impressive increase of the pCR rate in ER/PgR negative tumours with the weekly treatment could represent a simple ‘optical illusion’ if not followed by a substantial advantage in long-term disease-free survival of patients. An occult metastatic spread may derive from malignant cell clones, which have developed an acquired resistance to cytotoxic drugs, and are therefore less sensitive to the induction chemotherapy than the primary tumour. At the Mount Sinai Medical Center 144 patients with LABC treated with neoadjuvant chemotherapy were reviewed to evaluate the prognostic impact of chemotherapy-induced histological changes. Little evidence in favour of predictivity of pathological response was found (Gajdos *et al*, 2002). In another recent report from the US NCI, 107 patients with stage III breast cancer were treated with a multimodality approach. Pathologic response was not associated with improved survival for stage IIIA and inflammatory breast cancer patients (Low *et al*, 2004). In an MDACC retrospective analysis conducted on 372 women with LABC, the DFS and OS of the pCRs group were significantly better than those of the other group; however, investigators remarked that a pCR did not eliminate the risk of recurrence (Kuerer *et al*, 1999).

In the present study, a reduction of the risk of relapse with the PET regimen becomes evident after 3 years in ER/PgR negative patients, and is maintained beyond 5 years. Given the small study population, it is clear that such a difference cannot be statistically significant, even with a more mature follow-up. A new randomised trial, specifically targeted to ER/PgR negative patients, with an adequate sample size is required to address this issue.

The impressive increase of the pCR rate in HER2/neu positive patients with the weekly treatment also deserves some considerations. There are several possible factors responsible for the observed better tumour shrinkage in the PET arm. Firstly, the increase of the epirubicin dose intensity, as previously reported by French authors (Petit *et al*, 2001).

The adoption of a weekly paclitaxel schedule might also have a role. In the CALGB 9840 trial, (Seidman *et al*, 2004) weekly paclitaxel significantly improved ORR and survival of metastatic breast cancer patients, when compared with standard every 3 weeks paclitaxel. However, in patients with HER2/neu normal tumour, the weekly administration failed to produce a significant gain in both ORR and survival.

In spite of the very impressive pCR rate observed in HER2 positive patients, the DFS outcome was disappointing in this group, the risk of relapse being relevant even in pCRs. Of course, the discovery of a drug specifically targeting HER2/neu overexpressing tumour cells has deeply modified the therapeutic strategy in this subset of patients. The capability of trastuzumab in improving the prognosis of patients with metastatic disease is well established (Slamon *et al*, 2001). Preliminary reports also suggest that the addition of trastuzumab to a standard anthracycline-taxane combination can dramatically increase the pCR rate in women with operable breast cancer (Buzdar *et al*, 2005).

The addition of trastuzumab to a dose dense aggressive chemotherapy, like PET, could result in a further substantial increment of the pCR rate. Moreover, it could substantially increase the probability of obtaining the eradication of the distant micrometastases, as suggested by the results of several large randomised trials (Piccart-Gebhart *et al*, 2005; Romond *et al*, 2005).

A final consideration on the toxicity profiles of the two regimens deserves to be made. Although our dose dense approach did not cause any life-threatening toxicity, it produced a substantial increase of severe side effects, which may negatively impact on the quality of life of patients. Severe anaemia was significantly more frequent in the PET arm, and it resulted in a substantially higher proportion of patients complaining about severe fatigue. PET treatment was also associated with more severe nonhaematological toxicity. In particular, diarrhoea, peripheral neuropathy and mucositis occurred more frequently in the PET arm, being the main causes of patient's discomfort. In view of that, an accurate selection of patients who more likely can benefit of this approach is mandatory in the next future.

In conclusion, a 3-month preoperative treatment with weekly PET plus G-CSF support yields a significantly higher pathological complete response rate in women with LABC compared with four tri-weekly ET cycles. The superiority of the PET regimen was limited to hormone-receptor negative or HER2/neu overexpressing tumours. PET treatment was associated with a substantial increase in nonhaematological toxicity. A longer follow-up is needed to better evaluate the impact of this new approach on failure-free and OS. However, such a dose-dense approach is not recommended in women with ER/PgR positive LABC. Adequately powered, randomised trials are required to evaluate whether this approach can significantly improve prognosis of ER negative and/or HER2 positive LABC patients.

## Figures and Tables

**Table 1:** Demographics

**Characteristic**	**PET (100 patients)**	**ET (100 patients)**	**Total (200 patients)**
*Age*			
Median (range)	53 (27–73)	54 (30–72)	54 (27–73)
< 65/⩾65	52/48	49/51	101/99
			
*Stage*			
T4 N0	12	14	26
T4 N1	79	78	157
Any T N2	9	8	17
			
*Breast tumour*			
T4a-c/d	67/24	69/23	136/47
			
*Histology*			
Ductal	71	73	144
Lobular	19	18	37
Mixed	4	4	8
Mucinous	3	2	5
Other	3	3	6
			
*Grading*			
I	9	12	21
II	33	30	63
III	55	54	109
Unknown	3	4	7
			
*Menopausal status*			
Pre-/postmenopausal	33/67	31/69	64/136
			
*Hormone receptors status*			
ER: yes/no/unknown	53/40/7	57/37/6	110/77/13
PgR: yes/no/unknown	50/43/7	53/41/6	103/84/13
			
*HER/neu status*			
Pos/neg/unknown	23/68/9	20/68/12	43/136/21

**Table 2:** Clinical response according to treatment ARM

	**PET no. (%)**		**ET no. (%)**	**Total no. (%)**
Complete	28 (28)	*P*=NS	19 (19)	47 (23.5)
Partial	60 (60)		59 (59)	119 (59.5)
MR/NC	9 (9)		18 (18)	27 (13.5)
PD	3 (3)		4 (4)	7 (3.5)

**Table 3:** Complete response according to hormone receptors and her/neu status

	**PET no. (%)**		**ET no. (%)**	**Total no. (%)**
*Hormone receptors*				
ER−	19/40 (47.5)	*P*<0.05	6/37 (16.2)	25/77 (32.5)
ER+	8/53 (15)	*P*=NS	12/57 (21)	20/110 (18)
Unknown	1/7 (14.5)		1/6 (16.5)	2/13 (15.4)
				
*HER/neu*				
+	9/23 (40)	*P*=NS	7/20 (35)	16/43 (37.2)
−	16/68 (23.6)	*P*=NS	10/68 (14.7)	26/136 (19)
Unknown	3/9 (33.3)		2/12 (16.7)	5/21 (24)

**Table 4:** Pathological assessment according to treatment arm

	**PET no. (%)**		**ET no. (%)**	**Total no. (%)**
*Breast*				
Class I	13 (13)	*P*=NS	9 (9)	22 (11)
Class II	9 (9)	*P*=NS	5 (5)	14 (7)
Class III	38 (38)		26 (26)	64 (32)
Class IV	40 (40)		60 (60)	100 (50)
				
*Axilla*				
N0	35 (35)		24 (24)	59 (29.5)
N1–3	33 (33)		27 (27)	60 (30)
N >3	32 (32)		49 (49)	81 (40.5)
pCR (class I+II and N0)	16 (16)	*P*=0.02	6 (6)	22 (11)

**Table 5:** Pathological complete response according to pretreatment features

	**PET no. pCR (%)**	**ET no. pCR (%)**	**Total no. pCR (%)**
ER−	11/40 (27.7)*	2/37 (5.4)	13/77 (17)
ER+	4/53 (7.5)	4/57 (7.1)	8/110 (7)
Unknown	1/7 (14)	0/6	1/13 (8)
			
HER pos	7/23 (31)#	1/20 (5)	8/43 (18.6)
HER neg	7/68 (10)	4/68 (6)	11/136 (8)
Unknown	2/9 (22)	1/12 (8)	3/21 (14)

* PET *vs* ET: *P*=0.026.

# PET *vs* ET; *P*=0.037.

**Table 6:** Who grade 3–4 toxicity in the two arms

	**PET (100)**	**ET (100)**
	**No. of patients (%)**	**No. of patients (%)**
Neutropenia	45 (45)	38 (38)
Sepsis	3 (3)	4 (4)
Thrombocytopenia	4 (4)	2 (2)
Anaemia	13 (13)	1 (1)
Nausea	9 (9)	6 (6)
Vomiting	6 (6)	4 (4)
Loss of appetite	13 (10)	5 (5)
Diarrhoea	15 (15)	2 (2)
Mucositis	12 (12)	1 (1)
Neurotoxicity	3 (3)	0
Renal	0	0
Liver	0	0
Fatigue	11 (11)	2 (2)
		
*Cardiac toxicity*		
< 50% LVEF	5 (5)	3 (3)
Congestive failure	0	0
		
Skin toxicity	3 (3)	0
